# Possible protective effect of zinc administration on renal and cognitive changes occurring in uninephrectomized adult male Wistar rats

**DOI:** 10.1113/EP090735

**Published:** 2022-11-24

**Authors:** Marianne Basta, Hend A. Yassin, Rania G. Aly, Norhan S. El Sayed

**Affiliations:** ^1^ Department of Medical Physiology Faculty of Medicine University of Alexandria Alexandria Egypt; ^2^ Department of Medical Biochemistry Faculty of Medicine University of Alexandria Alexandria Egypt; ^3^ Department of Medical Pathology Faculty of Medicine University of Alexandria Alexandria Egypt

**Keywords:** BDNF, behaviour, desmin, GFAP, uninephrectomy, zinc

## Abstract

Cognitive impairment is increasingly recognized as an important consequence of kidney disease in humans. Kidney donation is a safe procedure but is known to increase the long‐term risk of cardiovascular and kidney disease. Whether kidney donation impairs cognitive function is not known. In the present study, we examined whether the renal changes occurring post‐nephrectomy were accompanied by cognitive changes as well, and whether early administration of zinc supplements such as ZnSO_4_ to uninephrectomized (UNX) rats could ameliorate the renal and cognitive changes if present. The present study included 30 adult male Wistar rats that were randomly assigned to three groups (*n* = 10 per group): sham‐operated rats, UNX and UNX treated with ZnSO_4_ for 20 weeks. Before termination, rats were subjected to 24‐h urine collection and behavioural testing with the Morris water maze and T maze tests. UNX induced significant proteinuria, renal functional, fibrotic and oxidative changes, as well as increased renal desmin expression. UNX rats also showed significant behavioural changes indicating spatial learning and memory affection, together with decreased hippocampal brain derived neurotrophic factor (BDNF) and antioxidant capacity, and increased glial fibrillary acidic protein (GFAP), nitric oxide and malondialdehyde. In addition, UNX induced significant hyperglycaemia and dyslipidaemia, as well as significant reduction in serum zinc, copper and selenium. Early administration of ZnSO_4_ starting 1 week post‐nephrectomy significantly ameliorated renal and behavioural changes, as well as hippocampal oxidative, BDNF and GFAP changes. Additionally, Zn recovered serum changes of triglycerides, cholesterol, zinc and copper. Therefore, early administration of zinc to humans undergoing nephrectomy may be of benefit and should be considered in human trials.

## Abstract (highlights)

- **What is the central question of this study?**
  Are renal changes occurring post‐nephrectomy accompanied by cognitive changes, and does early administration of zinc supplements such as ZnSO_4_ to uninephrectomized rats ameliorate the renal and cognitive changes if present?
- **What is the main finding and its importance?**
  Uninephrectomy‐induced renal changes were accompanied by species‐atypical behaviour in rats in both Morris water maze and T maze tests, together with hypozincaemia and hippocampal inflammatory and oxidative changes. Early zinc administration to uninephrectomized rats ameliorated the renal, behavioural, hippocampal and serum zinc changes.

## 1 INTRODUCTION

Living kidney donation has been the treatment of choice for patients with end‐stage renal disease (ESRD). Although kidney donation has been considered a safe procedure in healthy, low‐risk persons, it might have lifelong implications. Kidney donors showed increased risk of renal dysfunction that may progress to ESRD on the long term. Donors showed decreased glomerular filtration rate (GFR), marked albuminuria and increased serum creatinine as early as 6 months post‐nephrectomy. Albuminuria has been a well‐known marker of podocyte injury and renal disease progression (Roldán‐Reina et al., 2018). Reduced kidney function occurring post‐nephrectomy might generally affect other body systems, for example, general body metabolism (Zhao et al., 2008) and cardiovascular system (Moody et al., 2016).

Since rats respond at a faster rate to uninephrectomy (UNX), UNX rats have been used to mimic kidney donation in humans (Basta et al., 2019). Animal studies have demonstrated that UNX imposes a risk of developing chronic kidney disease. UNX rats showed no pathology or minimal changes up to 3 months post‐nephrectomy (Zhao et al., 2008), but afterwards, they developed renal structural and functional perturbations in the remaining kidney that might end with focal segmental sclerosis and ESRD, as well as cardiovascular changes and general metabolic abnormalities (Basta et al., 2019; Rodríguez‐Gómez et al., 2012).

Cognitive changes such as memory and learning disabilities are common in renal patients due to accompanying general body dyshomeostasis (Seliger et al., 2004) Similarly, 5 out of 6 nephrectomized rats showed deficits in spatial working memory and learning, as well as hippocampal inflammatory changes (Yu et al., 2022). Renal‐induced cognitive changes might result from the generalized body state of oxidative stress, metabolic disturbance, vascular dysfunction, electrolyte and acid imbalance, uraemia and inflammation (Viggiano et al., 2020). Many of these abnormalities have been also implicated in the renal impairment that occurs post‐nephrectomy (Basta et al., 2019; Moody et al., 2016; Rodríguez‐Gómez et al., 2012).

Yet little is known about the cognitive changes that might occur in patients undergoing UNX in the long term. As 1 month in a rat is equivalent to 3 years in humans, we have suggested that rats at 5 months post‐nephrectomy might show some cognitive changes. Therefore, this study has assessed the early cognitive changes that might occur in rats at 5 months post‐nephrectomy, which could be expanded to human studies later.

Zinc (Zn) is an essential trace element and an important antioxidant that maintains the integrity of the body's cellular functions. Zn deficiency is common in renal diseases due to increased renal loss, yet little is known about serum Zn levels following nephrectomy. Zn supplementation in rats with diabetic nephropathy showed protective effects on kidney structure and function with alleviation of proteinuria, fibrosis and renal oxidative stress (Piao et al., 2019; Tang et al., 2010; Zhang et al., 2016). Zn also has anti‐inflammatory and metabolic regulatory functions (Lobo et al., 2010). Additionally, low doses of zinc supplementation seem to exert a neuroprotective effect (Corona et al., 2010). Consequently, we have suggested that early Zn supplementation using zinc sulphate (ZnSO_4_) to UNX rats might help in preserving remaining kidney structure and function. We have also suggested that Zn may reverse serum Zn deficiency and cognitive changes that may occur in UNX rats.

Therefore, the aim of this study was to reveal the early cognitive changes that might occur in adult rats post‐nephrectomy and whether early administration of small doses of Zn supplement might modulate the early pathological renal changes reflecting nephroprotective and neuroprotective roles.

## 2 METHODS

### 2.1 Ethical approval

The study protocol was approved by the Research Ethics Committee of Alexandria Faculty of Medicine (IRB: 00012098; FWA: 00018699; serial no.: 0305058). The present study complied with the ARRIVE guidelines 2.0 regarding methods and animal handling. All precautions were taken to minimize animal pain and suffering.

### 2.2 Animals and husbandry

Thirty adult male Wistar rats (12–14 weeks old, 100–150 g body weight) were provided by animal house of the Physiology Department, Faculty of Medicine, Alexandria University. Rats had free access to standard laboratory chow and water and were kept under controlled 12‐h light–dark cycles. After 1 week of acclimatization, rats were weighed (Wt_0_) and randomly assigned to three different groups (*n* = 10 rats per group): control group, UNX group, and UNX plus Zn supplementation group (UNX+Zn).

### 2.3 Surgical procedures

UNX rats with or without zinc supplementation underwent surgical removal of the right kidney (Basta et al., 2019; Gai et al., 2014). Rats were weighed and anaesthetized using a combination of ketamine (75 mg/kg) and xylazine (10 mg/kg) administered by intramuscular injection. A right flank incision was made following anaesthesia. The right kidney was exposed and decapsulated, leaving the adrenal gland intact. The renal pedicle was ligated, the kidney was excised distal to the ligature, and the incision was sutured. Rats in the control group underwent sham surgery, exposing the right kidney without excision. Rats were injected immediately after surgery with 1 ml glucose and 1 ml saline intraperitoneally, and 1 mg/kg of the analgesic butorphanol subcutaneously. Rats received 20 mg/kg of the antibiotic ciprofloxacin subcutaneously, divided into two doses. Rats were placed on warm pads in dry, clean and comfortable cages. Paper towels were used for bedding to monitor bleeding. Regular clean bedding was returned the following day if there was no bleeding. Animals were monitored for any sign of distress for the following 3 days.

After 1 week of recovery, rats were weighed and administered daily either 15 mg ZnSO_4_ in 5 ml double‐distilled water per kilogram bodyweight (UNX+Zn group) or an equivalent amount of vehicle (control and UNX groups) via oral gavage daily till the end of the experiment (20 weeks post‐surgery) (Piao et al., 2019).

### 2.4 Behavioural tests

Just before termination of the study, all rats were subjected to the following behavioural tests.

#### 2.4.1 Modified Morris water maze

A modified Morris water maze (MWM) (Sterniczuk et al., 2010) task was performed by all rats. The test was done on four consecutive days. The water maze consisted of a dark circular pool, 125 cm in diameter, 55 cm in height, filled with opaque water (about 22 ± 3°C) to a depth of 20 cm. A submerged circular black platform (10 cm in diameter) was placed 20 cm away from the edge in a fixed location and 1 cm below the water surface. The pool was divided into four quadrants by four starting points marked on its wall: north–south–east–west. The platform provided the only escape from the water. Several cues were placed outside the maze in a fixed position relative to the pool: a window, coloured curtain and red flag; they helped the rat locate the position of the escape platform hidden below the water surface. The path taken by the animal was recorded by a digital video camera that was mounted above the centre of the pool to record the distance travelled and time taken by each rat to reach the platform. The distance was measured with the aid of a grid with 10 cm squares placed over the pool. Two steps were performed using the water maze task.

##### 2.4.1.1 Step 1

The aim of this step was to compare the performances of different groups during daily water maze training. It was performed on all experimental rats. Each rat received four trials per day for three consecutive days. Each rat was placed in the water facing the wall of the pool at one of the four designated starting points (north, east, south and west) and allowed to swim and find the hidden platform located in the NW quadrant (target quadrant) of the maze. Each of four starting positions was used once in four training sessions (Inostroza et al., 2011). During each trial, each rat was given 120 s to find the hidden platform. After mounting the platform, the rats were allowed to remain there for 20 s and were then placed in a holding cage for 30 s until the start of the next trial. After completion of training, the rats returned to their home cages. If the rats could not find the platform in 120 s, they were placed on it by the examiner and left there for 20 s. The time required for reaching the hidden platform (latency) was measured, and the distance travelled in the pool before reaching the platform was calculated from the recorded path taken by the rat. The time spent in the target quadrant was also measured (expressed as a percentage of the total time spent in the pool).

##### 2.4.1.2 Step 2

Twenty‐four hours after the last day of training for each group, memory retention testing was done to assess for memory consolidation. Retention testing consisted of 60 s of a free‐swimming period for each rat with the hidden platform removed from the pool. The better the memory is consolidated the more time will the rat swim around the location of the platform and fewer escape trials will be attempted from the wall of the pool. The time spent in the target quadrant (expressed as a percentage of the time spent in the pool, total of 60 s), the distance swum in the target quadrant (expressed as a percentage of the distance swum in the pool) and the number of escape trials from the edge of the pool were calculated.

#### 2.4.2 T‐maze alteration test

A T‐maze test using spontaneous alternation was used to evaluate working (short‐term) memory. The maze was set so that all guillotine doors were raised. The animal was placed in the start area and allowed to choose a goal arm. The rat was then confined in the chosen arm by quietly sliding the door down. After 30 s, the animal was removed. After that, the guillotine doors were raised, and the animal was replaced in the start area facing away from the goal arms. The rat was allowed to choose between the two open‐goal arms. Each trial should take no more than 2 min; 1 min is the minimum possible. Ten trials were done, and the percentage of correct alterations was calculated (Deacon & Rawlins, 2006).

### 2.5 Termination and tissue collection

Twenty weeks post‐surgery, rats were placed in metabolic cages with free access to food and water, and 24‐h urine was collected. On the day of termination, all rats were fasted overnight, weighed (terminal body weight, TBW), and killed using terminal anaesthesia. Rats were killed using a high dose of anaesthetic (200 mg/kg of ketamine and 10 mg/kg of xylazine) via intraperitoneal injection, and blood was collected by cardiac puncture (Xue et al., 2016). Left kidneys were harvested, weighed and their ratio to TBW was calculated. Half of the kidney was stored at −80°C for protein extraction and the other half was processed by paraffin‐embedding for histological examination (Basta et al., 2019). Similarly, the whole brain was removed and washed with ice‐cold saline, and one hemisphere was fixed with formalin for histological examination. The hippocampal tissues were quickly dissected from the other hemisphere and stored at −80°C for protein extraction and biochemical analysis (Inda et al., 2020). Homogenization of kidney and hippocampus was done on ice using RIPA buffer. The homogenates were centrifuged at 10,000 *g* for 10 min at 4°C. The supernatant was collected and stored at −80°C (Basta et al., 2019).

#### 2.5.1 Determination of the serum level of selenium, copper, zinc in rats by atomic absorption technique

The principle of the assay (Rostampour et al., 2018; Sabe et al., 1999) is as follows.

A graphite furnace atomic absorption spectrometer (GF‐AAS) (SavantAA, GBC Scientific Equipment, Chicago, USA), equipped with a PAL3000 auto sampler and GF5000 graphite furnace system, was used in all analyses. Deuterium background correction was used for selenium (Se) and Zn. For the determination of total selenium in rat serum using GF‐AAS, nickel nitrate as a chemical modifier was used.

Measurements were performed using a Se electrodeless discharge lamp (EDL) running at 10.00 mA, with a wavelength of 196.00 nm and a bandpass of 1.00 nm and a copper (Cu) EDL running at wavelength of 324.00 nm and Zn EDL running at wavelength of 312.00 nm. Argon with a purity of 99.996% was used as the carrier gas.

Absorbance was read at 196.0 nm. Peak height measurements were used for quantification. Non‐specific background absorption was avoided by conducting the deuterium background correction. A standard curve was drawn by Savant software where the absorbance value for each standard was plotted on the vertical axis against the corresponding standard concentration (μg/l) on the horizontal axis.

### 2.6 Biochemical studies

The Jaffe kinetic method was used to measure creatinine in serum and urine. The Berthelot enzymatic method was used to measure serum urea (Zhao et al., 2008). Reagent kits were obtained from Diamond Diagnostics (Hannover, Germany). The pyragallol red/SDS method was used to measure urine protein concentration (Watanabe et al., 1986). The reagent kit was provided by Wellkang Ltd (London, UK). All kits were used according to the manufacturers’ instructions.

Enzymatic methods were used to measure serum triglycerides, total and high‐density lipoprotein cholesterol (HDL‐C), and glucose in terminal samples. Kits were provided by BioSystems S.A. (Barcelona, Spain) and used according to the manufacturer's instructions. We used total cholesterol minus HDL‐C to calculate non‐HDL‐C (Basta et al., 2019).

#### 2.6.1 Determination of brain derived neurotrophic factor, total antioxidant capacity, nitric oxide and lipid peroxidation in hippocampal homogenates

Brain derived neurotrophic factor (BDNF) in hippocampal homogenates was detected by enzyme‐linked immunosorbent assay (ELISA) kit provided by Chongqing Biospe (Chongqing, China) according to the manufacturer's directions. It is based on sandwich ELISA technology. The duplicate readings for each standard, control and samples were averaged and the average zero standard optical density was subtracted. The absorbance at 450 nm was read with an ELISA reader (serial number: 315‐1414). A standard curve was created and the mean absorbance value for each sample was used to determine the corresponding concentration of BDNF in ng/ml (Huang et al., 2019).

Concentrations of total anti‐oxidant capacity, nitric oxide (NO) and malondialdehyde (MDA) were measured in the homogenate colourimetrically according to the assay kit protocol (BioDiagnostic, Egypt, Cairo) (Özcelik et al., 2012).

#### 2.6.2 Histopathological examination

Hippocampus and kidney specimens from the control, UNX and Zn‐treated groups were fixed in 10% buffered formaldehyde and embedded in paraffin blocks (FFPE). The sections from the FFPE blocks were stained with haematoxylin and eosin (H&E). The sections were examined using different magnifications of the light microscope (Leica Microsystems, Wetzlar, Germany).

##### Masson's trichrome staining

The nephrectomy FFPE blocks, from all groups, were stained with Masson's trichrome stain. ImageJ, colour deconvolution v1.53 (NIH, Bethesda, MD, USA) was used to quantify the percentage of fibrous tissue area per high‐power field in 10 microscopic fields (magnification was 100 μm as present on scale, and quantification was done in each microscopic field as a whole) (Chen et al., 2017). The estimation of glomerular affection was assessed on 50 glomeruli by renal cross‐section using the quantitative‐image analysis (Leica Microsystems).

##### Immunohistochemical staining and interpretation

Immunohistochemistry (IHC) of all sections was done using the avidin–biotin–peroxidase method using the Bond‐Max fully automated immunostainer (Leica Biosystems, Deer Park, IL, USA) (Hsu et al., 1981). GFAP (ready‐to‐use primary antibody, mouse anti‐human, monoclonal antibody, PA0026; Leica Biosystems) and desmin (ready to use primary antibody, mouse anti‐human, monoclonal antibody, PA0032; Leica Biosystems) were used to stain the hippocampus and renal glomerular and interstitial myofibroblasts, respectively. The antibodies were added to each section. The quantification of the IHC was done for each slide (five microscopic fields for GFAP and 20 microscopic fields for desmin; magnification was 50 μm as present on scale, quantification was done in each microscopic field as a whole), using quantitative‐image analysis (Leica Microsystems). The quantification of desmin in the glomeruli and interstitium was done using quantitative‐image analysis (Leica Microsystems).

Similarly, quantification of apoptotic cells in hippocampus required the addition of an apoptotic marker Anti‐Caspase‐3 antibody, monoclonal antibody (ab184787) which was quantified in five microscopic fields (magnification was 50 μm as present on scale, quantification was done in each microscopic field as a whole) using the quantitative‐image analysis (Leica micro‐systems, Switzerland).

### 2.7 Statistical analysis

Data are presented as means ± SD. We tested for normality using the Shapiro–Wilk test. Results were evaluated by one‐way ANOVA, followed by *post hoc* pair‐wise analysis without adjustment for multiple comparisons. Log‐10 transformations were made for skewed variables. All tests were two‐tailed, with *P* < 0.05 being considered significant. SPSS Statistics for Windows version 20.0 (IBM Corp., Armonk, NY, USA) was used for analysis.

## 3 RESULTS

### 3.1 Effect of ZnSO_4_ administration on weight gain, ratio of remaining kidney/TBW and renal functional changes in UNX rats

Both UNX and UNX+Zn groups showed significant reduction in TBW (≥10% reduction, *P* ≤ 0.01) and weight gain (TBW – Wt_0_, ≥20% reduction, *P* ≤ 0.01), compared to sham‐operated rats. On the other hand, the ratio of left kidney weight to TBW was significantly increased in UNX rats, compared to control rats (63% increase, *P* < 0.0001). However, ZnSO_4_ administration to UNX rats significantly ameliorated the increase in the ratio, compared to untreated rats (23% decrease, *P* < 0.0001).

Again, UNX rats showed significant increase in serum urea (38% increase, *P* < 0.0001) and creatinine (65% increase, *P* < 0.0001) levels, as well as urine protein/creatinine ratio (255% increase, *P* = 0.0016) and 24‐h proteinuria (159% increase, *P* = 0.0012) at 20 weeks post‐nephrectomy, compared to sham‐operated rats. Early administration of 15 mg ZnSO_4_/kg/day to UNX rats significantly ameliorated the serum changes of urea (11% decrease, *P* = 0.0246) and creatinine (15% decrease, *P* = 0.0461) levels, as well as the urine changes of protein/creatinine ratio (42% decrease, *P* = 0.0402) and 24‐h proteinuria (34% decrease, *P* = 0.0413), compared to untreated UNX rats (Table 1).

**TABLE 1:** Effect of zinc administration on body weight, metabolism and electrolytes, and kidney weights and functions in UNX rats

Variable	Control	UNX	UNX+Zn
TBW (g)	295.5 ± 7.7	265.5 ± 9*	259 ± 7.5*
Weight gain (g)	158 ± 6.6	126 ± 11*	117 ± 6*
Left kidney weight/TBW	0.32 ± 0.02	0.53 ± 0.02**	0.4 ± 0.02* ^,^ ^##^
Serum urea (mg/dl)	48 ± 3	67 ± 1.4**	60 ± 1.4 * ^,^ ^#^
Serum creatinine (mg/dl)	1 ± 0.06	1.68 ± 0.08**	1.43 ± 0.1* ^,^ ^#^
24‐h urine total protein (mg)	2.2 ± 0.2	5.7 ± 0.96*	3.7 ± 0.4^#^
Urine protein/creatinine ratio	1.1 ± 0.05	3.9 ± 0.9*	2.3 ± 0.16^#^
Serum zinc (ppb)	9.1 ± 1.1	3.7 ± 0.9**	7.4 ± 1.4^#^
Serum copper (ppb)	782.5 ± 59	379.4 ± 44*	600.5 ± 31^#^
Serum selenium (ppb)	89.5 ± 4	55.1 ± 1.5*	58.2 ± 5.3*
Fasting serum glucose (mg/dl)	69 ± 2.3	91.9 ± 4.4**	82 ± 1.8** ^,^ ^#^
Fasting serum triglycerides (mg/dl)	78.6 ± 2.9	114 ± 1.5**	96 ± 4.3* ^,^ ^#^
Fasting serum cholesterol (mg/dl)	76.4 ± 4.3	92.5 ± 3.6*	88.7 ± 4.6
Fasting serum HDL‐C (mg/dl)	59.2 ± 3	45.5 ± 2.6*	53 ± 1.5^#^
Fasting serum non‐HDL‐C (mg/dl)	17.2 ± 1.6	47 ± 2.8***	35.7 ± 4.8* ^,^ ^#^

Data are expressed as means ± SD. *n* = 10 per group. *P*‐values are from one‐way ANOVA, followed by *post hoc* pair‐wise analysis without adjustment for multiple comparisons. Log‐10 transformations were made for skewed variables.

^*^ *P* < 0.050 and ^**^ *P* < 0.001 versus control;

^#^ *P* < 0.05 and ^##^ *P* < 0.001, difference between UNX and UNX+Zn rats. Control, sham operated; UNX, uninephrectomized; UNX+Zn, uninephrectomized treated with zinc; TBW, terminal body weight.

### 3.2 Effect of ZnSO_4_ administration on serum electrolytes, glucose and lipid profile in UNX rats

UNX induced a significant decrease in serum values of Zn (59% decrease, *P* = 0.0002), Cu (52% decrease, *P*= 0.002) and Se (38% decrease, *P* = 0.003), compared to sham‐operated rats. Similarly, UNX induced significant changes in fasting serum glucose (33% increase, *P* = 0.0002), triglycerides (45% increase, *P* < 0.0001), total cholesterol (21% increase, *P* = 0.014), HDL‐C (23% decrease, *P* = 0.001) and non‐HDL‐C (169% increase, *P* < 0.0001) at 20 weeks post‐nephrectomy, compared to sham‐operated rats. Alternatively, ZnSO_4_ treatment significantly ameliorated the serum changes of Zn (100% increase, *P* = 0.002), Cu (58% increase, *P* = 0.044), triglycerides (16% decrease, *P* = 0.001), HDL‐C (16% increase, *P* = 0.045), and non‐HDL‐C (27% decrease, *P* = 0.033) compared to untreated UNX rats (Table 1).

### 3.3 Effect of ZnSO_4_ administration on renal fibrotic and desmin expression changes in UNX rats

**FIGURE 1:**
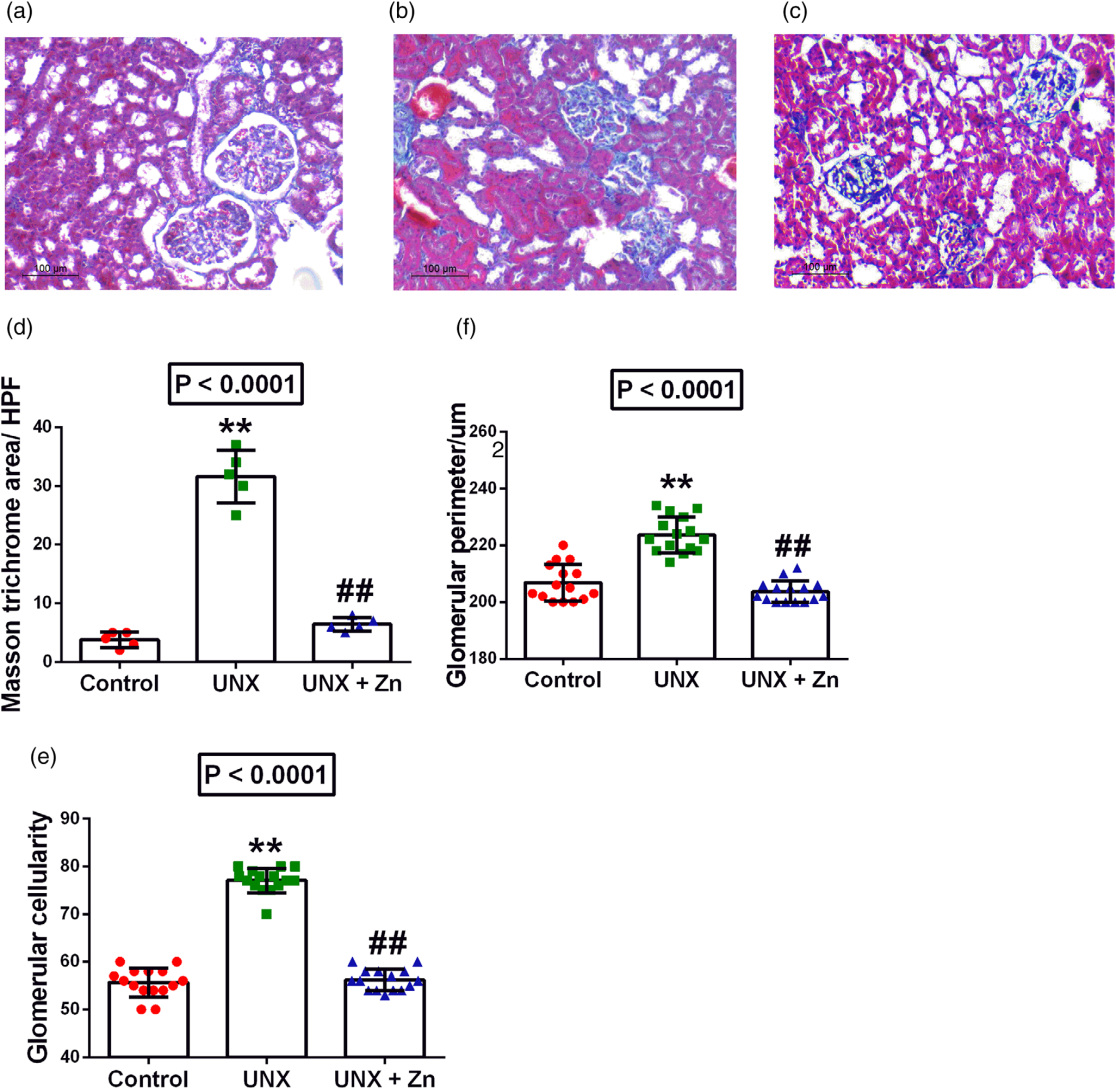
(a–c) Masson's trichrome staining of renal cortex and interstitium (original magnification ×200) at 5 months post‐surgery in control rats (a), uninephrectomized rats (UNX; b), and uninephrectomized rats with ZnSO_4_ treatment (UNX+Zn; c). (d–f) Image analysis of the findings shown in a–c: (d) Masson's trichrome area per high‐power field (HPF)); (e) glomerular cellularity; (f) glomerular perimeter. Data are means ± SD from *n* = 5 rats per group. *P*‐values are from one‐way ANOVA. **P < 0.001 relative to control; ##*P* < 0.001, significant difference between UNX and UNX+Zn rats

**FIGURE 2:**
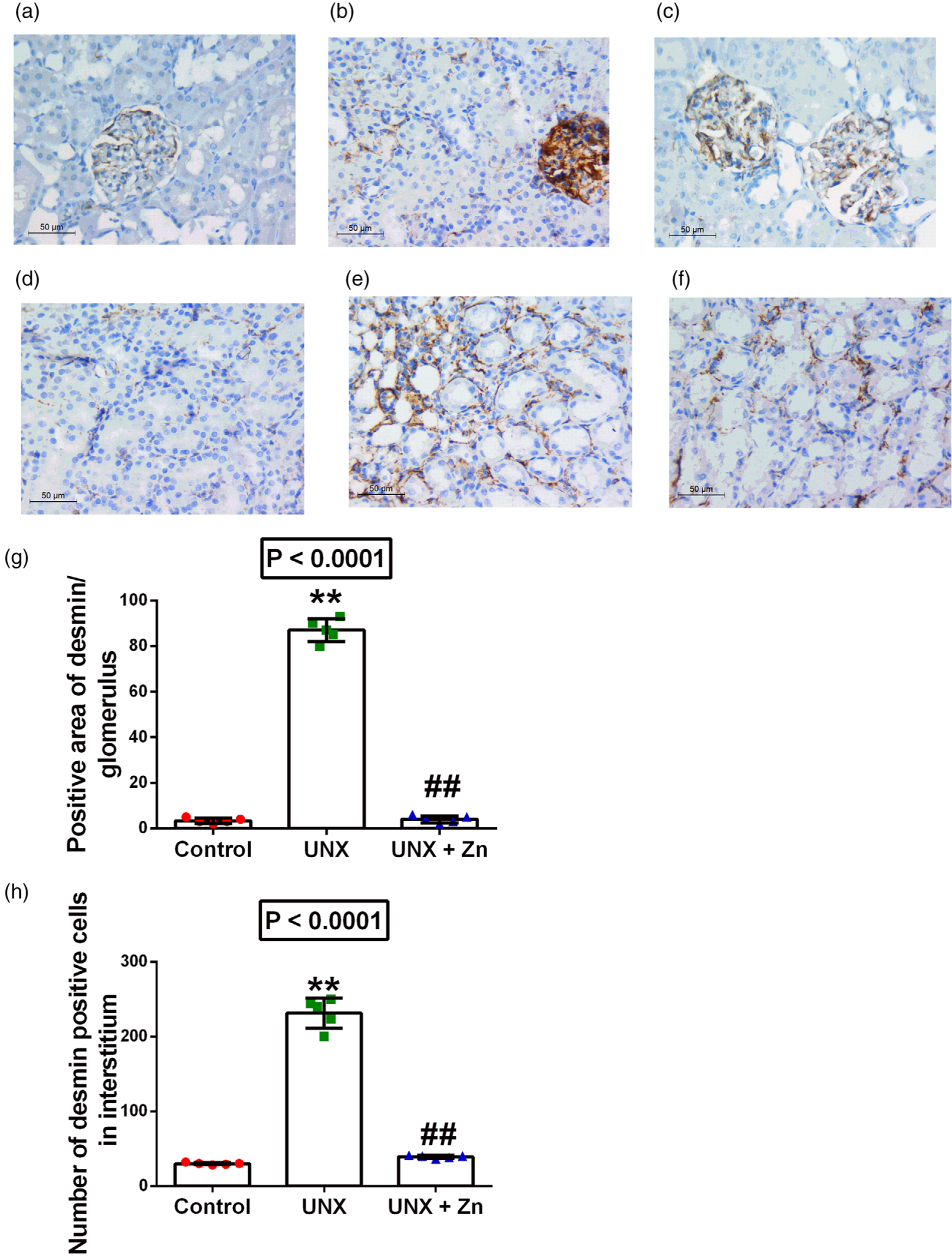
(a–f) Desmin immunostaining of renal glomeruli (a–c) and interstitium (d–f) (original magnification ×400) of control rats (a, d), uninephrectomized rats (UNX; b, e), and uninephrectomized rats with ZnSO_4_ treatment (UNX+Zn; c, f) at 5 months post‐surgery. (g, h) Analysis of the findings shown in a–f: (g) positive area of desmin/glomerulus; (h) number of desmin positive cells in interstitium. Data are means ± SD from *n* = 5 rats per group. *P*‐values are from one‐way ANOVA. **P < 0.001 relative to control; ##*P* < 0.001, significant difference between UNX and UNX+Zn rats

UNX induced significant glomerular hypertrophy manifested by increase in glomerular perimetry (8% increase, *P* < 0.0001) and cellularity (38% increase, *P* < 0.0001), compared to sham‐operated rats. Moreover, UNX induced significant glomerular and interstitial fibrotic changes (73% increase in Masson's trichrome stain area, *P* < 0.0001), together with increased desmin‐positive cells in both glomeruli (245% increase, *P* < 0.0001) and interstitium (670% increase, *P* < 0.0001), compared to control rats. However, early administration of Zn to UNX rats significantly ameliorated to near normal values the glomerular affection, fibrotic changes and desmin reactivity, compared to untreated rats (*P* < 0.0001). Although some tubular dilatation was seen in the Zn supplementation group, it was not of significance as it was localized rather than diffuse. Moreover, the full histological features of tubular injury such as oedema, tubular casts and vacuolization were not evident in this group, and there was no remarkable difference in tubular features in the three groups (Figures 1 and 2).

### 3.4 Effect of ZnSO_4_ administration on behavioural changes occurring in UNX rats

#### 3.4.1 Effect on learning and memory in Morris water maze

**FIGURE 3:**
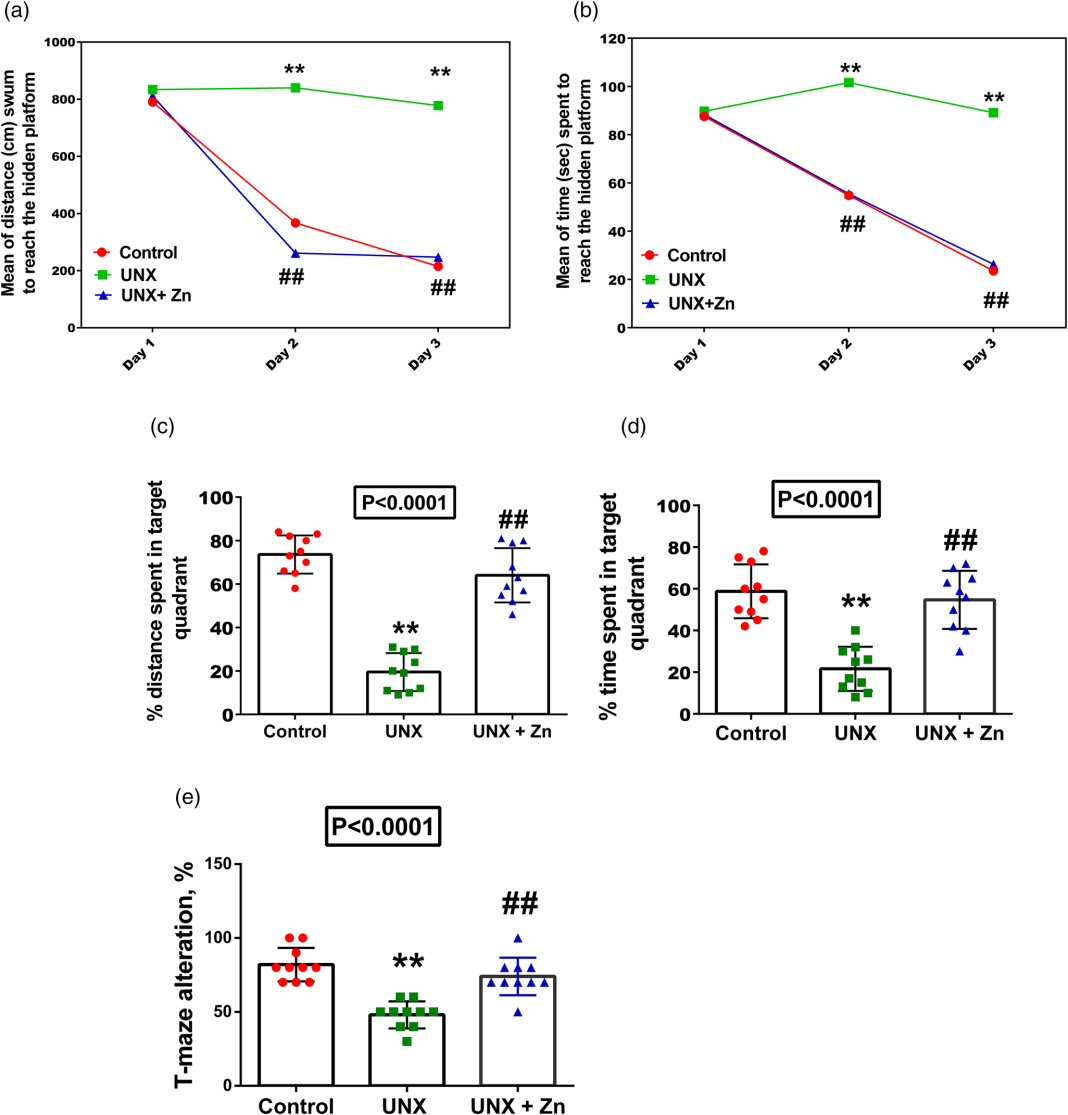
Behavioural tests in control rats, uninephrectomized rats (UNX), and uninephrectomized rats with ZnSO_4_ treatment (UNX+Zn) at 5 months post‐surgery. (a–d) Morris water maze. (a) Mean distance swum to reach the hidden platform. (b) Mean time spent to reach the hidden platform. (c) Percentage distance spent in target quadran. (d) Percentage time spent in target quadrant. (e) T maze alteration. Data are means ± SD, *n* = 10 rats per group. *P*‐values are from one‐way ANOVA. For skewed variables, log‐10 transformations were made. **P < 0.001 relative to control; ##*P* < 0.001, significant difference between UNX and UNX+Zn rats

Figure 3a and b shows spatial learning of the different study groups in the MWM. The average escape latency (the latency time to find the hidden platform) and escape distance (the path length to find the hidden platform) decreased with the increase in training days.

Untreated UNX rats showed a significant decrease in spatial learning, with longer latency and distance to reach the hidden platform. These results indicate that nephrectomy could significantly impair spatial learning and memory in rats (*P* < 0.0001). On the other hand, Zn‐treated rats showed a significant decrease in the meantime (latency) and the mean distance swum to reach the hidden platform, compared to the untreated UNX group (*P* < 0.0001) (Figure 3a and b).

In the memory retention test, control rats spent most time and swam most in the target quadrant demonstrating memory consolidation took place well in this group. Nevertheless, in the UNX group, time spent (37% decrease) and swimming distance (54% decrease) in the target quadrant were significantly less than those in the control group (*P* < 0.0001). On the other hand, treatment with Zn led to a significant increase in both the percentage of time (33% increase) and distance (44.5% increase) spent in the target quadrant, compared to the untreated UNX group (*P* < 0.0001) (Figure 3c and d).

#### 3.4.2 Effect on spatial memory in T maze

Spatial memory was found to be retarded in UNX rats as demonstrated by decreased spontaneous alternation in the T maze (34% decrease compared to the control, *P* < 0.0001). Meanwhile, Zn supplementation reverted percentage alteration to values near to controls (26% increase compared to untreated UNX rats, *P* < 0.0001) (Figure 3e).

### 3.5 Effect of ZnSO_4_ administration on hippocampal caspase‐3, BDNF and GFAP expression changes in UNX rats

**FIGURE 4:**
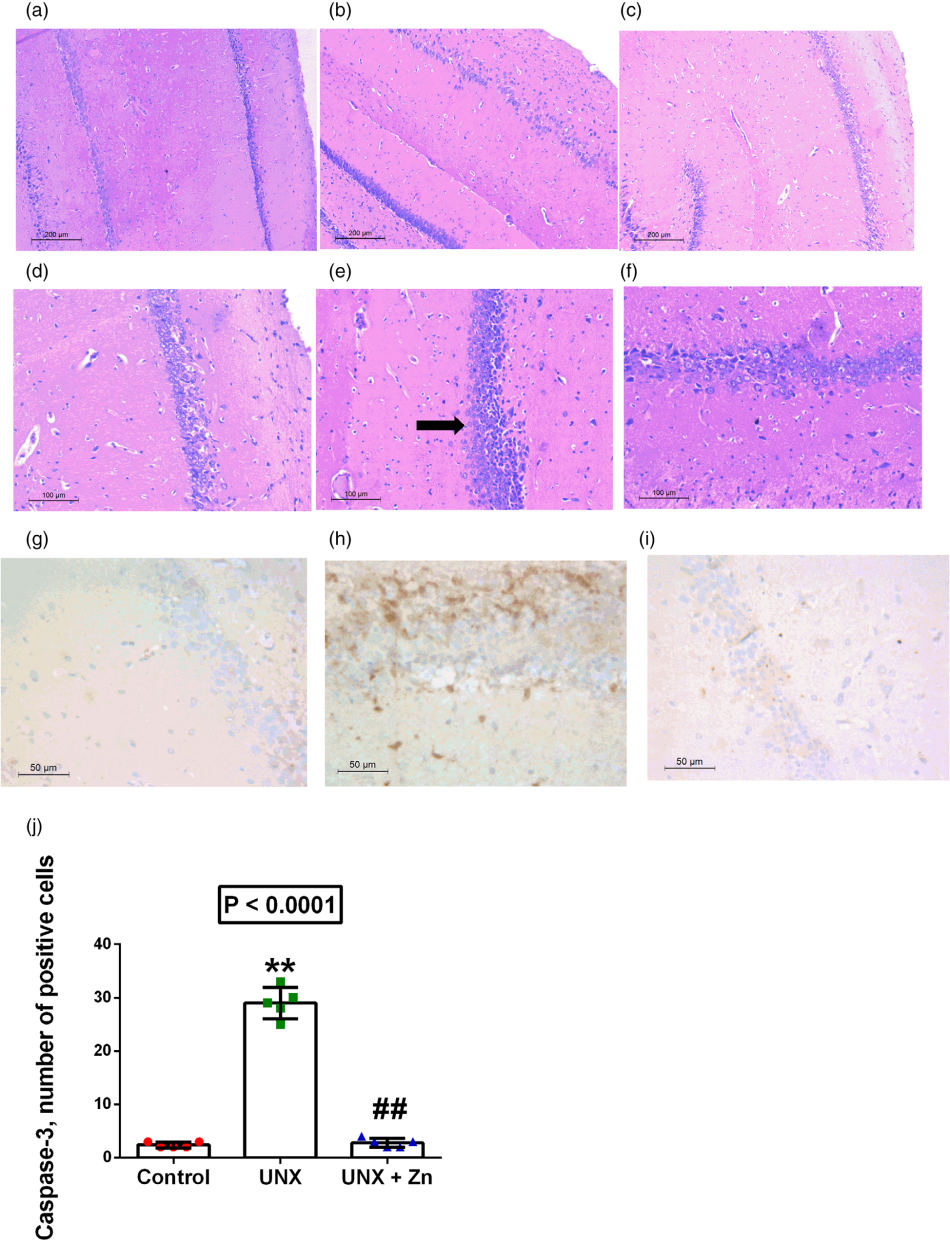
(a–f) Hippocampal apoptotic changes (a–c, original magnification ×100, d–f, original magnification ×200) at 5 months post‐surgery in control rats (a, d), uninephrectomized rats (UNX; b, e), and uninephrectomized rats with ZnSO_4_ treatment (UNX+Zn; c, f). (g–i) Caspase‐3 immunostaining of hippocampal tissues (original magnification ×400) at 5 months post‐surgery in control rats (g), uninephrectomized rats (UNX; h), and uninephrectomized rats with ZnSO_4_ treatment (UNX+Zn; i). (j) Image analysis of findings in (g–i): number of stained cells. Data are means ± SD from *n* = 5 rats per group. *P*‐values are from one‐way ANOVA. **P < 0.001 relative to control; ##*P* < 0.001, significant difference between UNX and UNX+Zn rats

UNX induced a significant increase in hippocampal apoptotic cells in H&E‐stained sections, as well as caspase‐3 positive stained cells (110% increase, *P* < 0.0001), compared to control rats. Alternatively, Zn treatment significantly ameliorated the hippocampal apoptotic process and decreased the number of caspase‐3 positive stained cells (90% decrease, *P* < 0.0001), compared to untreated rats (Figure 4).

**FIGURE 5:**
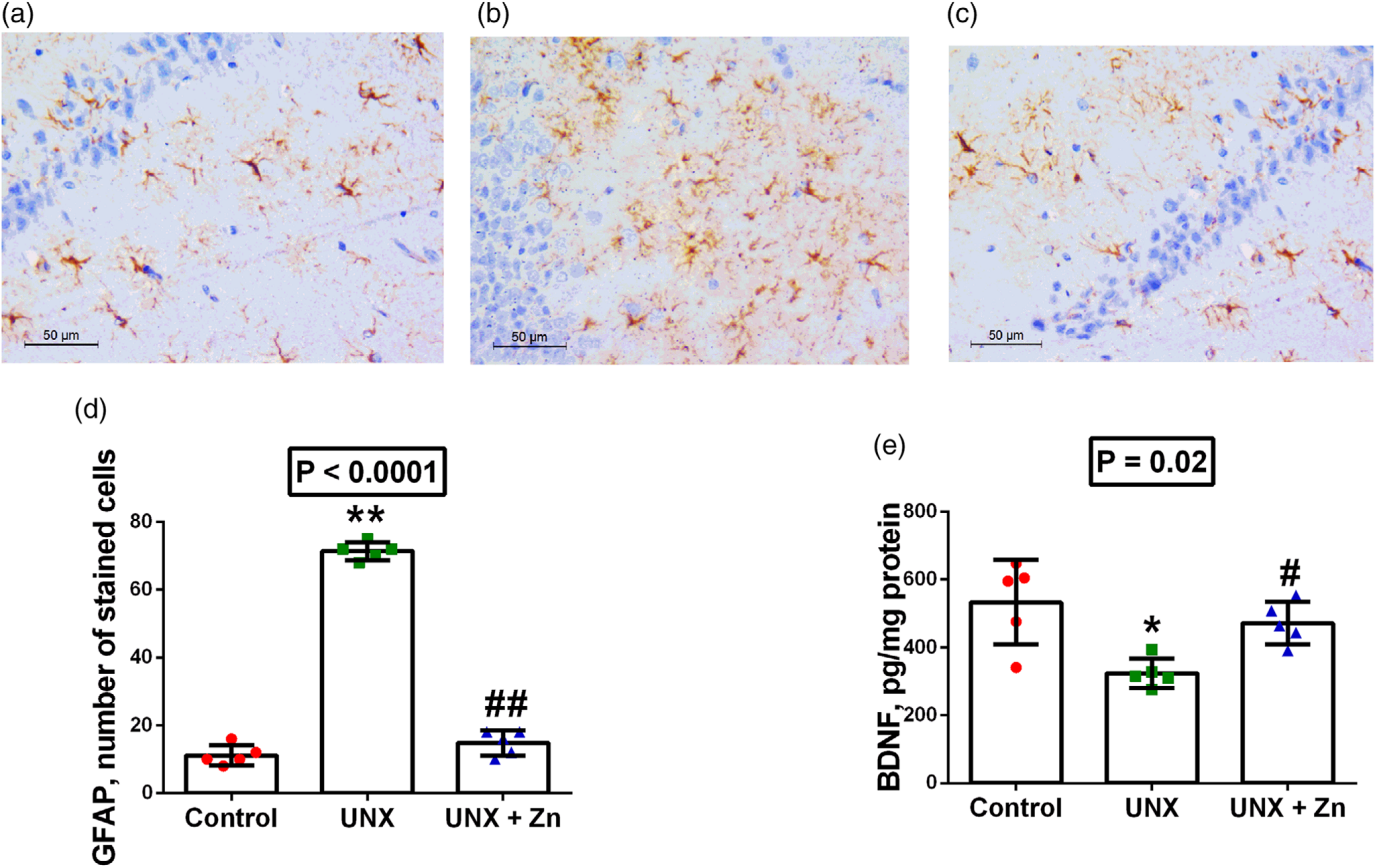
(a–c) GFAP immunostaining of hippocampal tissues (original magnification ×400) at 5 months post‐surgery in control rats (a), uninephrectomized rats (UNX; b), and uninephrectomized rats with ZnSO_4_ treatment (UNX+Zn; c). (d, e) Image analysis of findings in a–c: (d) number of stained cells; (e) BDNF concentration in hippocampal homogenates. Data are means ± SD from *n* = 5 rats per group. *P*‐values are from one‐way ANOVA. *P < 0.05, **P < 0.001 relative to control; #*P* < 0.05, ##*P* < 0.001, significant difference between UNX and UNX+Zn rats

Similarly, UNX induced a significant increase GFAP positive stained microglial cells (53% increase, *P* < 0.0001) in brain histological sections, compared to sham‐operated rats. However, ZnSO_4_ treatment significantly ameliorated hippocampal GFAP reactivity (79% decrease, *P* < 0.0001) in UNX rats, compared to untreated rats (Figure 5a–d). On the other hand, BDNF expression was significantly decreased in hippocampal homogenates of UNX rats (35% decreased compared to control rats, *P* = 0.008), while ZnSO_4_ treatment significantly upregulated it back to normal values (45% increase compared to untreated rats, *P* = 0.02) (Figure 5e).

### 3.6 Effect of ZnSO_4_ administration on hippocampal and renal oxidative profile changes in UNX rats

The total antioxidant capacity (TAC) was significantly decreased in both kidney (11% decrease, *P* = 0.013) and hippocampal (15% decrease, *P* = 0.004) homogenates of UNX rats, compared to sham‐operated ones. Early ZnSO_4_ administration to UNX rats significantly increased the TAC in both kidney (10% increase, *P* = 0.045) and hippocampal (19% increase, *P* = 0.003) homogenates of UNX rats, compared to untreated UNX ones (Table 2).

**TABLE 2:** Effect of zinc administration on hippocampal and renal oxidative profile changes in UNX rats

Variable	Control	UNX	UNX+Zn
Hippocampal TAC (mM/l)	1.29 ± 0.07	1.09 ± 0.1**	1.3 ± 0.08^#^
Kidney TAC (mM/l)	1.45 ± 0.08	1.29 ± 0.09*	1.41 ± 0.03^#^
Hippocampal NO (μmol/l)	25.2 ± 0.9	44.5 ± 4.6*	31.9 ± 5.2^#^
Kidney NO (μmol/l)	35.9 ± 2.7	44.6 ± 5.8**	36.9 ± 1.7
Hippocampal MDA (μmol/l)	247.3 ± 35	523.1 ± 47*	356.3 ± 43^#^
Kidney MDA (μmol/l)	544.5 ± 7.2	634.6 ± 26.2***	580.8 ± 10.8* ^,^ ^#^

Data are expressed as means ± SD. *n* = 10 per group. *P*‐values are from one‐way ANOVA, followed by *post hoc* pair‐wise analysis without adjustment for multiple comparisons. Log‐10 transformations were made for skewed variables.

^*^ *P* < 0.050, ^**^ *P* < 0.001 versus control;

^#^ *P* < 0.05, significant difference between UNX and UNX+Zn rats. Control, sham operated; UNX, uninephrectomized; UNX+Zn, uninephrectomized treated with zinc; MDA, malondialdehyde; NO, nitric oxide; TAC, total antioxidant capacity.

NO was significantly increased in both kidney (25% increase, *P* = 0.033) and hippocampal (75% decrease, *P* = 0.001) homogenates of UNX rats, compared to sham‐operated ones. Similarly, MDA was significantly increased in both renal (17% increase, *P* < 0.0001) and hippocampal (115% decrease, *P* = 0.003) homogenates of same rats. However, UNX rats treated with ZnSO_4_ showed significantly lower values for hippocampal NO (28% decrease, *P* = 0.013) and MDA (32% decrease, *P* = 0.038) and renal MDA (8% decrease, *P* = 0.001) compared to untreated UNX rats (Table 2).

## 4 DISCUSSION

The present study showed that UNX induced significant body weight reduction and compensatory renal hypertrophy as well as renal functional, structural and oxidative changes. The renal changes in UNX rats were accompanied by a significant decrease in serum levels of Zn, Cu and Se. UNX rats in the present study also demonstrated behavioural and cognitive changes, in addition to hippocampal apoptotic, oxidative and inflammatory changes. The renal oxidative, functional and structural changes, as well as metabolic changes occurring post‐nephrectomy were demonstrated in previous studies (Basta et al., 2019; Rodríguez‐Gómez et al., 2012; Zhao et al., 2008), yet the cognitive, behavioural and brain changes occurring post‐nephrectomy are not fully elucidated.

The renal functional changes included significant increase in serum urea and creatinine, as well as 24‐h proteinuria and urine protein/creatinine ratio at 5 months post‐nephrectomy. These changes were accompanied by renal fibrotic changes in histological sections as well as increased desmin expression in renal glomeruli and interstitium in kidney immuno‐stained sections. Enhanced staining of the intermediate filament protein, desmin, is a very sensitive marker of podocyte injury and progressive glomerular disease as demonstrated by Funk et al. (2016). Increased desmin production in glomeruli might have occurred due to mechanical stress imposed on podocytes by glomerular hypertrophy and hyperfiltration, and might indicate a state of epithelial‐to‐mesenchymal transition. Podocyte injury is commonly linked to proteinuria and fibrotic changes in renal diseases (Wiggins et al., 2005). The association between increased renal desmin expression and renal oxidative stress has been demonstrated before (Abou‐Zeid et al., 2021).

Renal homogenates also revealed increased production of NO and MDA and decreased TAC, indicating a renal ongoing oxidative process. Decreased serum levels of Zn, Cu and Se have been found to be common in CKD (Makhlough et al., 2015; Zachara, 2015). The reduced serum levels of Zn, Cu and Se in UNX rats might be due to the decreased renal tubular reabsorption (Damianaki et al., 2020; Makhlough et al., 2015), renin–angiotensin system activation (Thomas et al., 2007) and hypoalbuminaemia (Makhlough et al., 2015). The trace elements, Zn and Cu, as cofactors of superoxide dismutase and Se as a constituent of glutathione peroxidases, play an important role in the antioxidant defence system (Zachara et al., 2006). Therefore, decrease in serum levels of Zn, Cu and Se in UNX rats might explain the oxidative changes occurred in both kidney and hippocampus post‐nephrectomy. Moreover, decreased Zn might be related to hippocampal apoptotic changes as Zn has been shown to regulate both cell mitosis and cell death process of apoptosis (Truong‐Tran et al., 2000).

The renal changes were accompanied by significant increase in fasting serum glucose, triglycerides, total cholesterol and non‐HDL‐C, and significant decrease in HDL‐C. Dyslipidaemia is common in renal dysfunction and can aggravate the progression of chronic kidney disease. Lipid dysmetabolism induced renal lipotoxicity, oxidative stress, inflammation, disruption of glomerular structure, interstitial fibrosis and proteinuria (Pei et al., 2020). Renal dysfunction associated dyslipidaemia has been an important risk factor of atherosclerotic, cardiovascular and brain insults that appear in renal patients. Zn deficiency, which commonly occurs in renal dysfunction, has been considered one of the potential causes of renal dyslipidaemia and atherosclerosis (Lobo et al., 2010).

Early administration of Zn to UNX rats significantly ameliorated the renal functional, structural and oxidative changes. Few studies have addressed the importance of administration of reno‐protective agents to UNX rats. A previous study of resveratrol administration to UNX rats showed similar suggesting protective effects of resveratrol on the remaining kidney (Basta et al., 2019). Zn treatment also upregulated serum level of Zn and Cu to near normal values, and ameliorated UNX induced dyslipidaemia. Derouiche et al. showed that ZnSO_4_ administration to diabetic rats offered protection against renal oxidative and functional changes imposed by diabetes (Derouiche et al., 2017). Moreover, Özcelik et al. showed that ZnSO_4_ administration to diabetic rats preserved normal renal structure and histology, as well as renal function. They related its nephro‐protective role to stimulation of metallothionein synthesis and regulation of the oxidative stress, promoting an antioxidant effect and decreasing lipid peroxidation (Özcelik et al., 2012). Zn also downregulated kidney expression of pro‐fibrotic growth factors, pro‐inflammatory and pro‐apoptotic proteins that increase in renal diseases (Piao et al., 2019).

Significant behavioural changes were demonstrated post‐nephrectomy in both MWM and T maze, suggesting a long‐term negative impact of UNX on spatial learning and memory. The behavioural changes were accompanied by significant changes in hippocampal expression of BDNF and GFAP, as well as oxidative markers. Zn treatment ameliorated the behavioural and hippocampal oxidative changes and restored hippocampal BDNF and GFAP to near normal values.

Behavioural changes demonstrated in UNX rats could be attributed to decreased serum levels of Zn. Behavioural changes and cognitive impairment could occur in animals and humans suffering from Zn deficiency. Normally, Zn is present in high amounts in the hippocampus, which is considered as the area of learning and memory. Adult rats suffering from Zn deficiency showed impaired memory and decreased capacity to learn (Hagmeyer et al., 2015). Also, Zn plays an important role in synaptic plasticity, modulates synaptic transmission and is important for both intracellular and intercellular neuronal signalling. Zn is present in high concentrations in synaptic vesicles of the so called ‘Zn containing neurons’ and controls synaptic excitability by modulating the release of both glutamate and GABA. Moreover, Zn acts as a component of the catalytic site of almost 300 enzymes, most of which are present in the CNS (Tardy et al., 2020). Moreover, hypozincaemia, demonstrated in UNX rats, might be the cause of the decreased anti‐oxidative reserve in brain, which might lead to blood–brain barrier disruption and neuronal affection (Ebuehi & Akande, 2009).

Behavioural changes demonstrated in UNX rats may be also linked to the ongoing hippocampal oxidative stress, neurotoxicity and degeneration (Mazumder et al., 2019). We demonstrated increased hippocampal apoptotic activity, manifested by increased caspase‐3 expression in immunostained sections. This could be related to the increased MDA, NO and decreased TAC activities in hippocampal homogenates, which revealed an ongoing oxidative process in hippocampus, leading to spatial learning impairment (Nicolle et al., 2001). Neurons have been particularly vulnerable to oxidative stress because of increased O_2_ utilization, the relatively poor availability of classical antioxidants and related enzymes, and the high concentration of polyunsaturated lipids (Ebuehi & Akande, 2009). Moreover, NO shared in the production of the harmful free radical factor, peroxynitrite, which has been found to be responsible for oxidative and nitrosative pathways in neurons (Najafi et al., 2013).

Another cause for the revealed oxidative stress might be increased serum urea, which might cause the accumulation of uraemic toxins in different brain regions (Reza‐Zaldívar et al., 2020). The UNX‐induced dysmetabolism with the manifested hyperglycaemia and dyslipidaemia might be another reason for UNX‐induced brain oxidative changes (Pei et al., 2020).

Moreover, the behavioural changes might be related to changes in hippocampal BDNF and GFAP expression, which are common in renal dysfunction (Ma et al., 2022; Mazumder et al., 2019). BDNF decrease and GFAP increase could be related to the inflammatory and oxidative changes occurring in hippocampus, as well as urinary Zn losses (Kida et al., 2015; Lima Giacobbo et al., 2019). Consecutively, Zn treatment restored BDNF expression and GFAP immunoreactivity in hippocampus of UNX rats to near normal values. BDNF is a neurotrophic factor for neurons responsible for memory and cognitive performance, and plays a modulatory role in neuronal plasticity like long‐term potentiation of learning (Gärtner & Staiger, 2002). Zn induces BDNF expression and potentiates its signalling pathway through activation of metalloproteinases, which convert pro‐BDNF to mature BDNF (Hwang et al., 2011). On the other hand, GFAP is secreted by astrocytes and considered as a marker of astrocytosis in neurodegenerative disorders (Oeckl et al., 2019).

### 4.1 Conclusion

UNX was associated with significant renal fibrotic and functional changes and proteinuria, as well as increased renal desmin expression, indicating an ongoing process of podocyte injury and glomerular disease. UNX rats also showed significant behavioural changes in both the MWM and T maze, revealing spatial learning and memory affection. UNX rats also showed a significant increase in hippocampal apoptotic cells and GFAP reactivity, and significant decrease in hippocampal BDNF expression. Early administration of Zn to UNX rats significantly ameliorated the renal, behavioural and hippocampal apoptotic changes, and restored hippocampal BDNF and GFAP to near‐normal values. Therefore, we recommend early administration of Zn to UNX patients kidney donation, for its nephro‐ and neuro‐protective effects.

### 4.2 Limitations of the study

We showed changes in species‐specific behaviour in the MWM and T maze and focused on hippocampal changes in UNX rats. However, other changes might happen in other brain parts, manifesting as changes to the animal's behaviour that we did not test. Again, serum Zn level might affect the activity of many enzymes and molecules that might play a role in the present study that we have not investigated yet.

## AUTHOR CONTRIBUTIONS

Marianne Basta and Norhan Sobhy established the study design, conducted the animal model and behavioural tests, and were responsible for data collection and analysis. Hend A. Yassin ran the biochemical measurements, ELISA and electrolyte analysis. Rania G. Aly conducted the histopathological examination and immunostaining, with analysis and interpretation. All authors contributed to preparing the manuscript and revisions. The authors declare that all data were generated in‐house and that no paper mill was used. All authors have read and approved the final version of this manuscript and agree to be accountable for all aspects of the work in ensuring that questions related to the accuracy or integrity of any part of the work are appropriately investigated and resolved. All persons designated as authors qualify for authorship, and all those who qualify for authorship are listed.

## CONFLICT OF INTEREST

The authors declare that they have no competing interests.

## FUNDING INFORMATION

No funding was received for this work from a specific funding body.

## Supporting information

Statistical Summary Document

## Data Availability

All data are available from the corresponding author.
